# Comparing Dimerization Free Energies and Binding Modes of Small Aromatic Molecules with Different Force Fields

**DOI:** 10.3390/molecules26196069

**Published:** 2021-10-07

**Authors:** Ilias Patmanidis, Riccardo Alessandri, Alex H. de Vries, Siewert J. Marrink

**Affiliations:** 1 Groningen Biomolecular Sciences and Biotechnology Institute and Zernike Institute for Advanced Materials, University of Groningen, Nijenborgh 7, 9747 AG Groningen, The Netherlands; patmanidis.ilias@gmail.com (I.P.); ric.alessandri@gmail.com (R.A.); A.H.de.Vries@rug.nl (A.H.d.V.); 2 Pritzker School of Molecular Engineering, University of Chicago, Chicago, IL 60637, USA

**Keywords:** dimerization free energy, force field comparison, small aromatic molecules

## Abstract

Dimerization free energies are fundamental quantities that describe the strength of interaction of different molecules. Obtaining accurate experimental values for small molecules and disentangling the conformations that contribute most to the binding can be extremely difficult, due to the size of the systems and the small energy differences. In many cases, one has to resort to computational methods to calculate such properties. In this work, we used molecular dynamics simulations in conjunction with metadynamics to calculate the free energy of dimerization of small aromatic rings, and compared three models from popular online servers for atomistic force fields, namely G54a7, CHARMM36 and OPLS. We show that, regardless of the force field, the profiles for the dimerization free energy of these compounds are very similar. However, significant care needs to be taken when studying larger molecules, since the deviations from the trends increase with the size of the molecules, resulting in force field dependent preferred stacking modes; for example, in the cases of pyrene and tetracene. Our results provide a useful background study for using topology builders to model systems which rely on stacking of aromatic moieties, and are relevant in areas ranging from drug design to supramolecular assembly.

## 1. Introduction

Determining the binding energies between molecules is of major importance in different scientific fields, such as drug design or nanotechnology [1,2]. The strength and dynamics of the interactions will determine whether a specific binding will take place or not, how long it will last and how it affects its surroundings. Experimental techniques, such as nuclear magnetic resonance [3,4,5] or isothermal titration calorimetry [6,7], can be used to calculate binding energies between macromolecules, but they are limited by the nature and the size of the molecules, since small energy differences are extremely difficult to be captured. Additionally, experimental techniques measure ensemble averages without detailed knowledge of the contributions of the different accessible conformations that they are averaged over. As a result, detailed experimental data are scarce and are usually available for large systems, such as large aromatic compounds or proteins, where the differences in energy can be measured with better accuracy and confidence [3,8].

Computational approaches comprise a powerful tool to measure the interaction strength and binding modes between small molecules, and are usually employed to support experimental measurements or deal with experimental pitfalls [9]. Their level of detail and computational complexity vary, and many different methods have been developed that are more or less suitable for different applications. Specifically, while quantum mechanical (QM) calculations in vacuum provide an accurate description for the favorable binding modes, the size of the systems and the need for treating the solvent explicitly render the use of QM methods prohibitively expensive in systems aiming to represent realistic environments [10,11]. On the other hand, docking simulations are a fast option to study the interactions between two molecules, but the simplifications in scoring functions and the limited information on the dynamics of the interactions restrict their use to modeling protein-ligand binding [12,13,14]. In molecular dynamics (MD), Newton’s equations of motion are solved to obtain trajectories of atomistic systems and study their physical and chemical properties. MD simulations can provide high resolution atomistic descriptions and capture the dynamic behavior of the systems under study. They are an ideal option to study binding energies and modes, since they combine high level of accuracy and reasonable computational costs [15,16,17,18].

In MD simulations, the combination of the mathematical models and the parameters that are used to describe the interactions among all the atoms of each system is called force field. A simple functional form of a force field includes two terms that describe the interactions between atoms that are covalently bonded (bond lengths, angles, and dihedral terms) and the interactions between non-bonded atoms (van der Waals forces and electrostatics), Equation (1).

(1) $$\begin{array}{c} E=\\ E_{bonded}+E_{non-bonded}=\\ E_{bonds}+E_{angles}+E_{dihedrals}+E_{LJ}+E_{Coulomb} \end{array}$$

bonds and angles are treated as harmonic oscillators, whereas dihedral angles are represented either as harmonic oscillators or periodic functions. Interatomic interactions (attractive dispersion and Pauli repulsion) are modeled with Lennard–Jones (LJ) potentials and electrostatic forces with Coulomb potentials.

Several force fields have been developed over the past decades, AMBER [19], CHARMM [20], GROMOS [21], OPLS [22], etc. Their functional forms are very similar, but there are some variations depending on the simulation program [23]. Furthermore, each force field was parametrized with a different strategy regarding amongst others, the order in which parameters were defined, fixed, and refined, the combination rules, the number of atom types and modularity, as well as the choice of specific experimental or QM results for calibration, and it was optimized to reproduce properties of specific systems, e.g., proteins, lipids, etc. Despite their differences, most force fields are sufficiently accurate and can be used in different applications [24,25]. It should also be kept in mind that the force fields used in this study are intended to be used without extensive reparametrization in a wide range of biomolecular systems, and are evolving as more pertinent experimental data become available; in particular free energy partitioning data were not used in the first parametrization, but have been incorporated in the last two decades. For some force fields, these aspects have been addressed in the literature [26,27].

A common issue that is encountered in MD simulations is finding and optimizing parameters for different systems, in order to make sure that the model represents an accurate description of the actual system and reproduces its physical properties. Nowadays, there are several web servers that generate parameters for MD simulations on demand for different types of molecules [28,29,30], broadening the accessibility of computational methods to novice users and making simulations of exotic or novel molecules plain sailing. Even if parameters for MD simulations can be easily obtained, there are two important limitations of MD simulations that affect every system under study. First, reactivity of molecules is not described in classical MD simulations, since covalent bonds cannot break or form. This means that the protonation state of the molecules remains the same and catalysis cannot be studied with conventional simulations. Second, there is no treatment for the electronic polarization, since atoms have fixed partial charges. In reality, the partial charges of atoms are dynamic and the electron density of the molecules changes based on its surroundings. Potential solutions to these limitations are the use of polarizable force fields [31] and combined QM/MD approaches [32], respectively, but both options come at the expense of computational cost. Other issues are related to the size of systems and the attainable simulation time scales. However, recent developments in coarse-grained methods showed that even though the atomistic resolution is reduced, the important structural and dynamical features of different molecules can be recollected, thus pushing the limits of molecular simulations and their applications [33,34,35].

**Figure 1:**
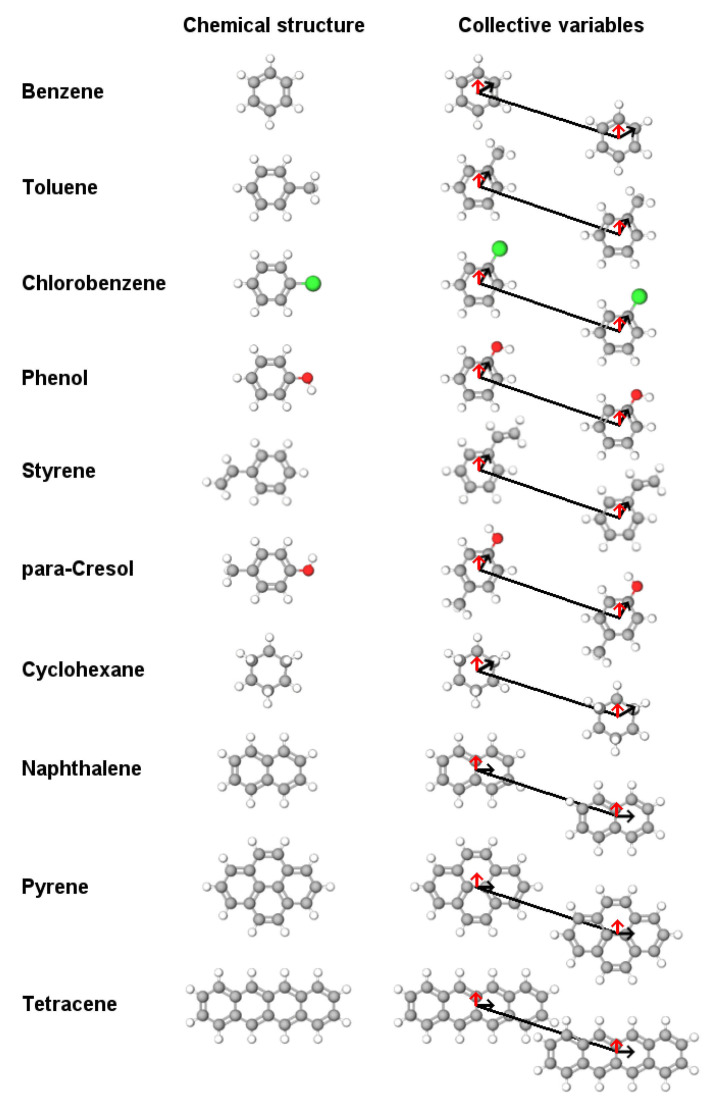
List of the molecules with their chemical structure considered in this work. The black line connects the center of geometry (COG) of the molecules and together with the black vectors represent the dihedral angle for the relative orientation of the dimers. The red vector denotes the normal of the plane for each molecule. The dihedral between the two black vectors and the angle between the red vectors, together with the COG distance, were used as collective variables (CV) for the calculation and presentation of the FES profiles.

In this study, we compared the dimerization free energy surface (FES) and binding modes for a series of small aromatic molecules (Figure 1) between three commonly used MD force fields: CHARMM36 [36], G54a7 [37] and OPLS [22]. These molecules are usually encountered as solvents (e.g., benzene or cyclohexane) or as building blocks of biomolecules and nanomaterials. Previous MD studies showed that different force fields produce consistent results in terms of structural and energetic properties, and they are usually compatible with QM or experimental studies, when available [10,38,39]. Our study focuses on small aromatic molecules and shows that, regardless of the force field, the free energy of binding and the binding modes are roughly the same. However, applications involving larger aromatic molecules should be considered with care. Increasing the size of the molecules leads to significant deviations in the FES profiles.

## 2. Results

### 2.1. Comparison of Parameter Setups

**Figure 2:**
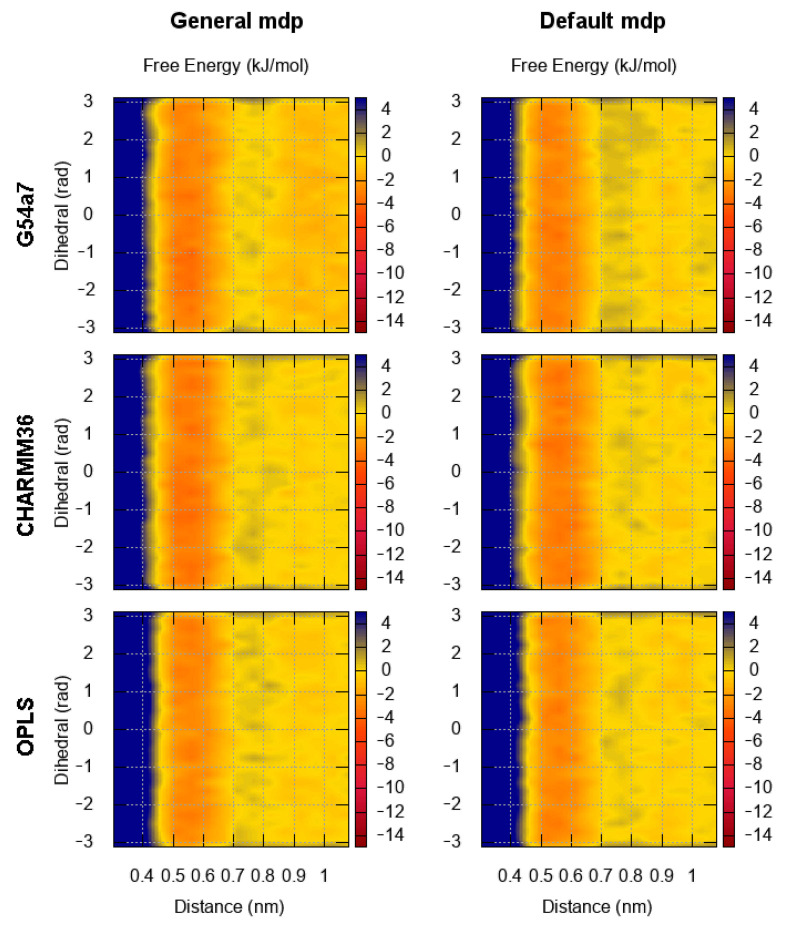
FES for the dimerization of benzene as a function of the distance and the torsional angle. The results for each force field are presented in each row. The plots on the left correspond to the FES based on the general setup and the plots on the right to the default parameters of each force field (see Methods).

**Figure 3:**
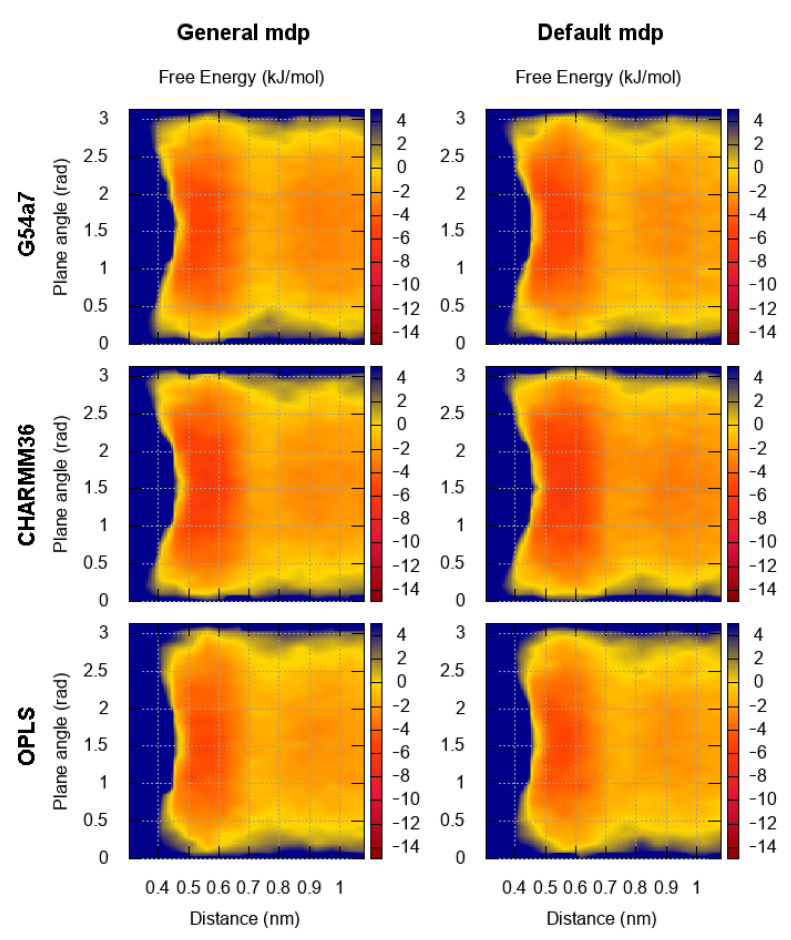
FES for the dimerization of benzene as a function of the distance and the angle between the normal of benzene planes. The results for each force field are presented in each row. The plots on the left correspond to the FES based on the general setup and the plots on the right to the default parameters of each force field.

**Figure 4:**
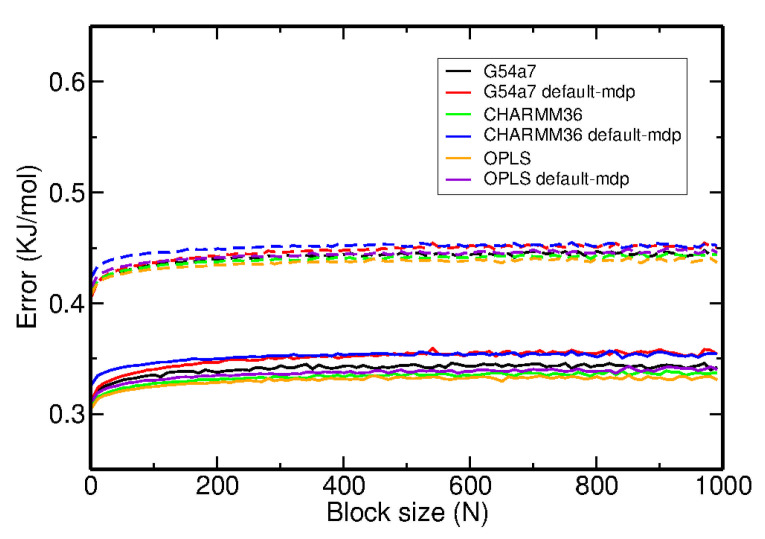
Statistical error of the FES calculations as a function of the block size. The solid lines correspond to the error analysis of the original biased simulation, whereas the dashed lines correspond to the reweighted profiles.

Benzene, the simplest aromatic compound, was used as a reference system for comparing the results between different force fields and studying the effect of using the default cut-off parameters or the general setup in the FES of dimerization. The results for the FES as a function of the distance between the center of geometry (COG) of two benzenes and the dihedral angle describing the relative orientation are presented in Figure 2. In Figure 3, the FES as a function of the distance between the COG and the angle between the normal of each benzene ring is presented. Figure 2 and Figure 3 show that the FES for the dimerization of benzene is roughly the same in all simulations. The average error of all points of the FES as a function of the block size is presented in Figure 4. The average error does not change as a function of the block size, indicating that the simulations were converged.

Regardless of the simulation setup, the binding energy and modes in the first interaction shell (∼0.4–0.7 nm) are almost identical. The only noticeable difference is seen in the G54a7 case, where the well in the second interaction shell (∼0.8–1.1 nm) is deeper ∼1–2 kJ/mol in the general setup compared to the simulation with the default parameters. Such differences might be proven important in some applications, but, as discussed later, we are mostly interested in the strength of binding and the orientation in the first interaction shell.

### 2.2. Comparison of FES Profiles

Next, we compare the binding modes of different aromatic molecules among different force fields. Starting with benzene, there is no preference in its orientation with respect to the dihedral angle, since benzene is symmetrical. However, conformations with angles ∼90${}^{\circ }$ (T-shaped) are more favorable than stacked conformations. When the FES is projected on the distance and the dihedral angle, the energy minimum in the first interaction shell is ∼3 kJ/mol, whereas the depth of the well is ∼5 kJ/mol, when the angle between the normal of the planes is used. In the first case, conformations with low and high energy minimum are grouped together, resulting in a lower value for the energy, whereas in the second case, the main variable that discriminates between the two conformations is effectively dissociated. This effect is only visible when the proper collective variables (CV) are used for projecting the FES. Nevertheless, our results for the FES of benzene are close to the reported values from previous Monte Carlo [40] and MD simulations [40], where the depth of the well was ∼6 kJ/mol and ∼1.5 kJ/mol, respectively, and the minimum of the FES was located at distance ∼0.55 nm in both studies.

**Figure 5:**
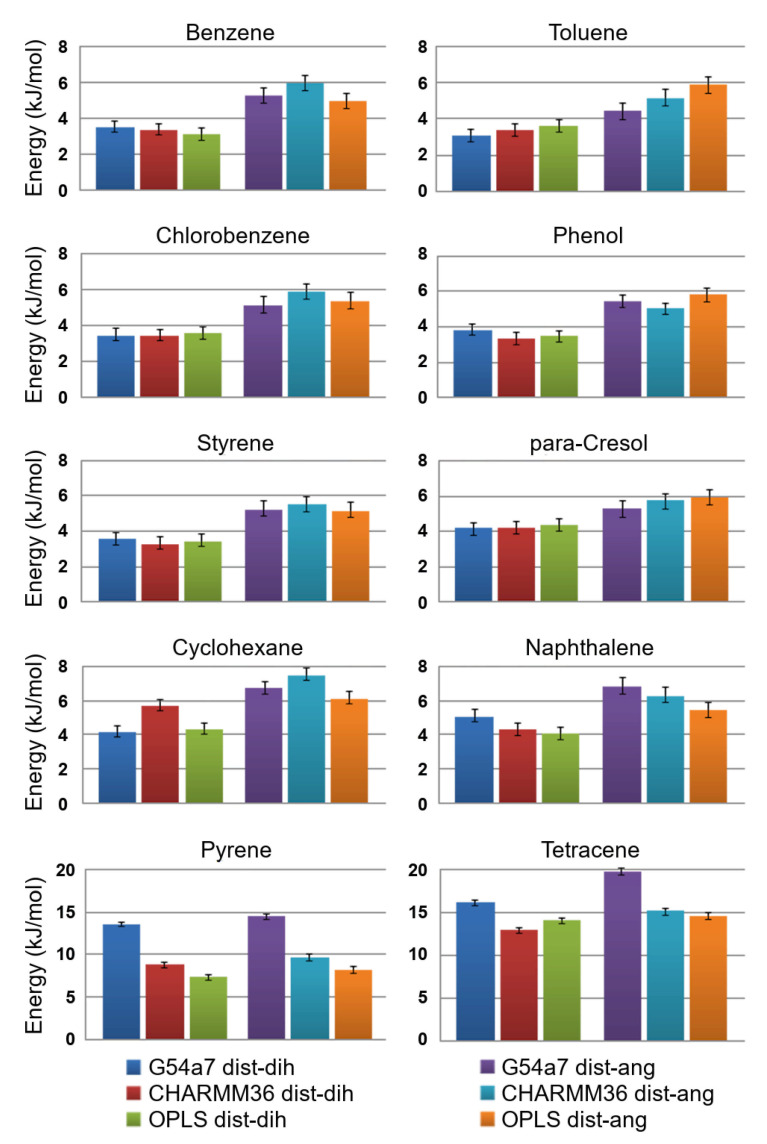
Binding energy at the lowest point of each molecule’s dimerization FES for different force fields. All simulations were performed with the general setup. The error bars correspond to the average error calculated from block analysis.

Regardless of the force field, similar results are obtained for most of the aromatic rings regarding the depth of the minimum energy in the first interaction shell, Figure 5. The energy differences among them are in the order of thermal fluctuations (<*k*${}_{b}$*T*), suggesting that their energy states are almost indistinguishable.

The FES profiles for each molecule and the standard errors are included in the Supplementary Material Figures S1–S7. T-shaped conformations are mainly preferred by most of the aromatic rings. At the same time, conformations close to the stacked arrangement are more populated in some molecules, e.g., chlorobenzene, as shown in Figure S2. Surprisingly, molecules with polar groups do not show significant differences in the orientation of the binding modes with respect to the dihedral angle that could be attributed to the presence of the charged groups. For example, phenol has a polar hydroxyl group that, in principle, should favor conformations where the oxygens would be diametrically opposed. However, this polar effect of the hydroxyl group was not observed in any FES, Figure S3. Such behavior might have been captured by using a polarizable force field. Effects in the orientation of the molecules with respect to the dihedral angle become slightly visible in para-cresol, Figure S5, and get more evident as the size of the ring increases.

**Figure 6:**
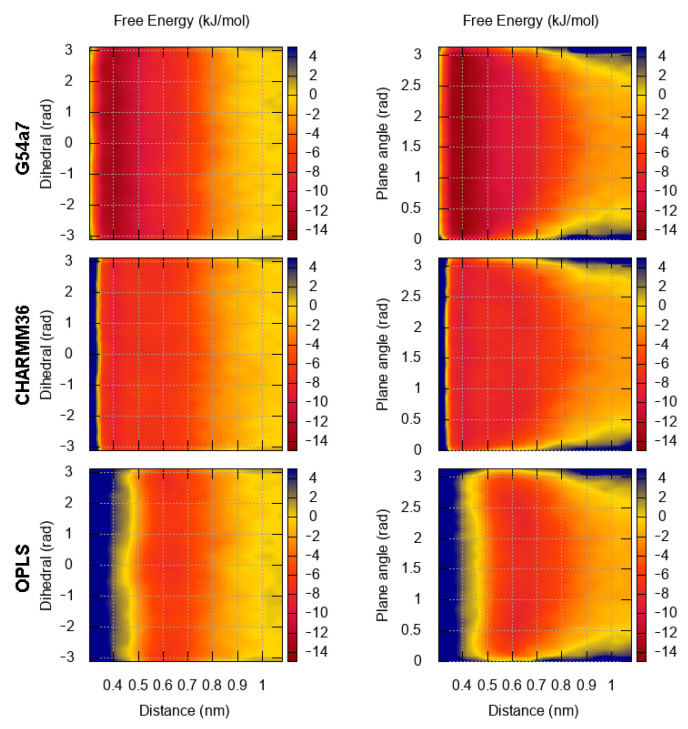
FES for the dimerization of pyrene as a function of the distance and the torsional angle (**left**) and the distance and the angle between the normal of aromatic planes (**right**).

**Figure 7:**
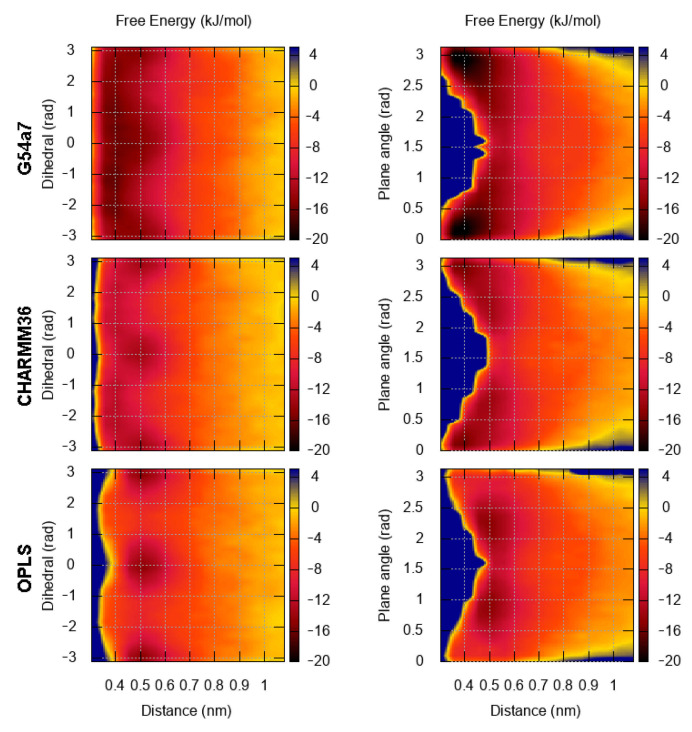
FES for the dimerization of tetracene as a function of the distance and the torsional angle (**left**) and the distance and the angle between the normal of aromatic planes (**right**).

Even though the results for the small aromatic molecules are roughly the same, as the size of the aromatic moiety increases, so do the deviations between each force field, Figure 5. The most extreme differences are observed in the FES of pyrene and tetracene, Figure 6 and Figure 7. For pyrene, the estimated energy of binding is ∼5 kJ/mol higher in the G54a7 simulation and the position of the minimum is located at ∼0.4 nm, suggesting that a sandwich conformation is preferred. On the other hand, there is no preference in the orientation of the molecules when they are at close proximity and minor differences in the orientation are only seen in the OPLS simulation. For tetracene, G54a7 predicts that parallel and close arrangements are more favorable, whereas according to CHARMM36, parallel arrangements and conformations with angle ∼0–60${}^{\circ }$ are similar in terms of energy. The difference in the energy of binding between G54a7 and CHARMM36 is ∼5 kJ/mol in the sandwich conformation. Finally, OPLS predicts that conformations with angle ∼30–90${}^{\circ }$ are the most favorable and the energy of binding is similar to CHARMM36. Deviations at the distance at which the minimum energy is found are present in both pyrene and tetracene FES profiles. The error estimation for these calculations are included in the Supplementary Material Figure S8.

## 3. Discussion

Obtaining accurate experimental results for binding energies of small molecules is usually difficult, and calculating such properties in realistic environments with high degree of accuracy using computational methods is quite expensive. Most computational studies for the interactions of aromatic rings are, therefore, obtained in the gas phase, or by investigating binding within a limited amount of degrees of freedom. A quick evaluation of the reported values in the literature shows that computational results are dependent on the method of use [10,41,42], and present deviations ∼2–4 kJ/mol for small aromatic molecules. Consequently, we consider that comparisons are mostly meaningful within specific methods, e.g., QM or MD, where the setups are controlled, the complexity and degrees of freedom are similar and deviations from the trends can be easily monitored. Taking this point into account, we only focused on the position and the depth of the well in the first interaction shell.

Our measurements for the dimerization free energy of small aromatic molecules suggest that different force fields produce similar results in terms of binding energies and modes. The presence of deviations in larger aromatic molecules could be attributed to different causes. First, most servers generate parameters based on similarity of atoms or molecules to specific databases of existing optimized molecules. Thus, the decrease in accuracy is inevitable, when the size and complexity of the target molecule increases. Even when the parameters are generated on the fly, the size and complexity of the molecules are important factors, and the models should be used with extra caution. The method for obtaining the parameters in each force field could be another reason for the small differences in the position and strength of binding. Such systematic differences are likely to accumulate when the size of the molecules increases. For example, the method for obtaining partial charges is reported to have significant effects on different thermodynamic properties, such as the free energy of hydration [43]. Additionally, these values might differ slightly or considerably among different force fields [44]. Despite the small differences in the FES profiles, the parameters seem to converge, at least for the small aromatic rings of this study.

To summarize, we performed MD simulations of small aromatic molecules to study the FES of their dimerization. Different force fields were used to compare the obtained FES and examine their convergence. The scarce experimental and computational results in similar environments make comparisons between experiments and theory, or different theories, extremely difficult. Thus, performance can be only assessed within the boundaries of MD simulations. Our results show that, regardless of the force field, we obtained free energy profiles with similar binding modes and strength of interactions. This enhances our confidence in using MD simulations to calculate the binding affinity of small compounds. On the other hand, deviations from the general trends were observed in large aromatic molecules, such as pyrene and tetracene, indicating that further optimization of the force fields is needed, before using such computational methods to make accurate quantitative predictions for large systems. This task is demanding and requires many iterations to guarantee that the target data from either QM calculations (e.g., vibrational spectra, dipole moments, etc.) or experimental measurements (e.g., thermodynamic properties, crystallographic data, etc.) have been effectively translated to MD parameters and the models are accurate descriptions of the underlying systems. In the future, machine learning methods could be the answer to the complex task of force field parameter refinement [45].

## 4. Methods

### 4.1. Molecule Parameters

We conducted MD simulations to study the FES for the dimerization of small aromatic molecules and compare the obtained FES from different force fields. The parameters for all the simulated molecules were obtained from different online servers that generate topology and parameter files for each force field. Specifically, the automated topology builder (ATB) server [28] (https://atb.uq.edu.au/, accessed on 1–15 November 2018) was used for the GROMOS based G54a7 [37] simulations. ATB includes a large database of topologies for different molecules. In the cases in which optimized parameters for the molecules of interest were available, those parameters were chosen. The ATB entries are reported in the Supplementary Material, Table S1. The CGenFF server (https://cgenff.umaryland.edu/, accessed on 1–15 November 2018) was used to generate the CHARMM36/CGenFF [29] parameters, whereas for the OPLS [22] parameters, the LigParGen server [30,46,47] (http://zarbi.chem.yale.edu/ligpargen/, accessed on 1–15 November 2018) and the 1.14*CM1A-LBCC charge model were used [47].

### 4.2. MD Simulations

All simulations and analysis were performed with Gromacs.2016-2018 [48,49] packages patched with PLUMED 2.4 [50]. Each simulation was performed in a cubic box with dimensions of approximately 5×5×5 nm, containing two of the selected molecules. The temperature was kept constant at 300 K with the v-rescale algorithm and a time constant of 0.1 ps [51]. For the pressure coupling, the Berendsen barostat [52] was used to maintain the pressure constant at 1 bar in an isotropic pressure bath with a time constant of 1 ps and compressibility of 4.5×10${}^{-5}$ bar${}^{-1}$. The cut-off for electrostatic and van der Waals interactions was set to 1.4 nm and the Verlet scheme was used for the short range non-bonded interactions with the default buffer tolerance of 0.005 kJ mol${}^{-1}$ ps${}^{-1}$. Long range interactions were calculated with the reaction field method [53] and $\epsilon_{r}$ set to 54. The LINCS algorithm was employed for constraining the bond lengths [54]. All systems were minimized for 10${}^{3}$ steps by using the steepest descent algorithm and equilibrated in the NVT and NPT conditions, each for 10 ps with a 2 fs time step. The production phase lasted 100–150 ns depending on the system, until the average errors in the free energy calculations from the block analysis converged. System details are included in the Supplementary Material Table S1.

Modeling water is not trivial and each force field was optimized with specific water model parameters to reproduce experimental and theoretical results. In order to be consistent with the original work and obtain the same accuracy, the appropriate water model should be used with each force field. Consequently, G54a7 systems were solvated with explicit SPC water molecules [55], whereas CHARMM36 and OPLS systems were solvated in TIP3P water model [56].

Apart from the water models, there are additional parameters that guarantee optimal performance when using each force field. In our case, we used the same cut-offs and parameters regardless of the force field to maintain simplicity and be consistent in the way the simulations were performed. This setup is referred to as general parameters. At the same time, we performed test simulations with setups as close as possible to the default parameters of each force field to evaluate the sensitivity of the results between the two setups. In the default G54a7 parameters, the group cut-off scheme was used with a twin range cut-off for the non-bonded interactions, where the short-range neighbor cut-off distance was set to 0.8 nm and the long range cut-off to 1.4 nm. In the CHARMM36 simulations, the LINCS algorithm was applied only to hydrogen atoms, the Verlet scheme was used with 1.2 nm distance cut-off and the long range electrostatic interactions were calculated with the particle mesh Ewald (PME) method [57]. As for the OPLS systems, the Verlet scheme was used with 1.0 nm distance cut-off and the PME method was used for the long range electrostatic interaction.

Finally, to stay updated with the latest halogen parametrization and compare potential effects in the FES, the chlorobenzene for the CHARMM36 force field was modeled with a Drude oscillator around the chlorine to model the effect of the “sigma hole” [58,59], a small area opposite to the bond that has positive electrostatic potential. The charge of the Drude particle was set 0.05 e and its mass to 0.1 u. The bond length between the Drude particle and the chlorine was 0.164 nm and the force constant 2508 kJ mol${}^{-1}$ nm${}^{-2}$. The equilibrium value for the angle formed by the carbon of the benzene ring, the chlorine and the Drude particle was set to 180${}^{\circ }$ and the force constant to 836 kJ mol${}^{-1}$ rad${}^{-2}$.

### 4.3. Metadynamics

The FES profiles for the dimerization of each molecule were obtained by metadynamics simulations [60,61] with the well-tempered [62] adaptation. In metadynamics, also known as local elevation [63] or conformational flooding [64], positive Gaussian potentials are added on the system as a memory dependent factor that drives the system towards unexplored regions of the available conformational space. After the simulation, the deposited Gaussians are summed and they are used to calculate the unbiased free energy landscape as a function of the CVs of interest. In the well-tempered adaptation, the height of the deposited Gaussian is smoothly decreased leading to converged FES profiles. The CVs on which the bias was added were the distance between the COG of the aromatic rings and the torsional angle between atoms of the rings and their COGs, Figure 1. The angle between the normal of the ring planes was used as a third CV to project the FES by using a reweighting algorithm [65]. The height of the deposited Gaussians was set to 1.0 kJ/mol and the width to 0.05 nm and 0.2 rad for the distance and torsion, respectively. Gaussians were deposited every 500 steps and the bias factor was set to 5. A wall, in the form of a harmonic potential with force constant 200 kJ/mol, was added at distance beyond 2 nm to prevent the molecules from exploring conformations at distances beyond the second interaction shell. All profiles were translated in order to be zero at distances where the profiles were almost flat (∼1.7 nm). Block analysis was used to estimate the error from the free energy calculations.

### 4.4. Entropic Component of Free Energy

Since we are dealing with molecules in a multi-dimensional space, the FES profiles should be corrected for the entropic component of the free energy. Imagine an object in a three dimensional space and another object that can be placed around the first one on a sphere of specific radius. In this case, the probability of finding the second object in a specific distance increases with the surface of the sphere. Because the probability of finding an object increases at larger distances, the free energy of interaction should be modified as a function of distance. The contribution of the entropic term in the FES has been taken into account when calculating and presenting the profiles by adding an additional energy component to the FES. The formula for the entropic component is shown in Equation (2).

(2) $$∆V_{FES}\left(r\right)=\left(n-1\right)\;R\;T\;\ln\left(r\right)$$

where ∆*V*${}_{FES}$(*r*) is the entropic free energy as a function of distance (*r*), *n* is the number of dimensions, *R* is the gas constant and *T* is the temperature of the system.

## Data Availability

Data is contained within the article or supplementary material.

## Supplementary Materials

The following are available, Table S1: ATB entries for the topologies used. Most of the parameters have been optimised to reproduce experimental solvation free energies, Figure S1: FES for the dimerization of toluene (top) and statistical error of the FES calculations as a function of the block size (bottom), Figure S2: FES for the dimerization of chlorobenzene (top) and statistical error of the FES calculations as a function of the block size (bottom), Figure S3: FES for the dimerization of phenol (top) and statistical error of the FES calculations as a function of the block size (bottom), Figure S4: FES for the dimerization of styrene (top) and statistical error of the FES calculations as a function of the block size (bottom), Figure S5: FES for the dimerization of para-cresol (top) and statistical error of the FES calculations as a function of the block size (bottom), Figure S6: FES for the dimerization of cyclohexane (top) and statistical error of the FES calculations as a function of the block size (bottom), Figure S7: FES for the dimerization of naphthalene (top) and statistical error of the FES calculations as a function of the block size (bottom), Figure S8: Statistical error of the FES calculations as a function of the block size for pyrene and anthracene, respectively.
