# Supplementary material for: Comparing Dimerization Free Energies and Binding Modes of Small Aromatic Molecules with Different Force Fields
Source: Molecules. 2021 Oct 7;26(19):6069. doi: 10.3390/molecules26196069 (PMC8512883; doi:10.3390/molecules26196069)
Supplement: Supplementary file 1 [file molecules-26-06069-s001.zip › molecules-1393609-supplementary.pdf]

Supplementary Material for

Comparing dimerization free energies and binding modes of small aromatic molecules with different force fields

Ilias Patmanidis,[1] Riccardo Alessandri,[1,2] Alex H. de Vries,[1] Siewert J. Marrink*[1]

[1] *Groningen Biomolecular Sciences and Biotechnology Institute and Zernike Institute for Advanced Materials, University of Groningen, Nijenborgh 7, 9747 AG Groningen, The Netherlands*

[2] *Pritzker School of Molecular Engineering, University of Chicago, Chicago, Illinois 60637, United States*

E-mail: s.j.marrink@rug.nl

Name	Molecule ID	Opt. Solvation Free Energy	Simulation time (ns)
Benzene	804	✔	100
Toluene	21	✔	100
Chlorobenzene	3176	✔	150
Phenol	1061	✔	100
Styrene	1266	✔	100
para-Cresol	17	✔	150
Cyclohexane	17666	✔	100
Naphthalene	820	✔	100
Pyrene	7233	✔	150
Tetracene	33259	✗	150

Table S1: ATB entries for the topologies used. Most of the parameters have been optimised to reproduce experimental solvation free energies.

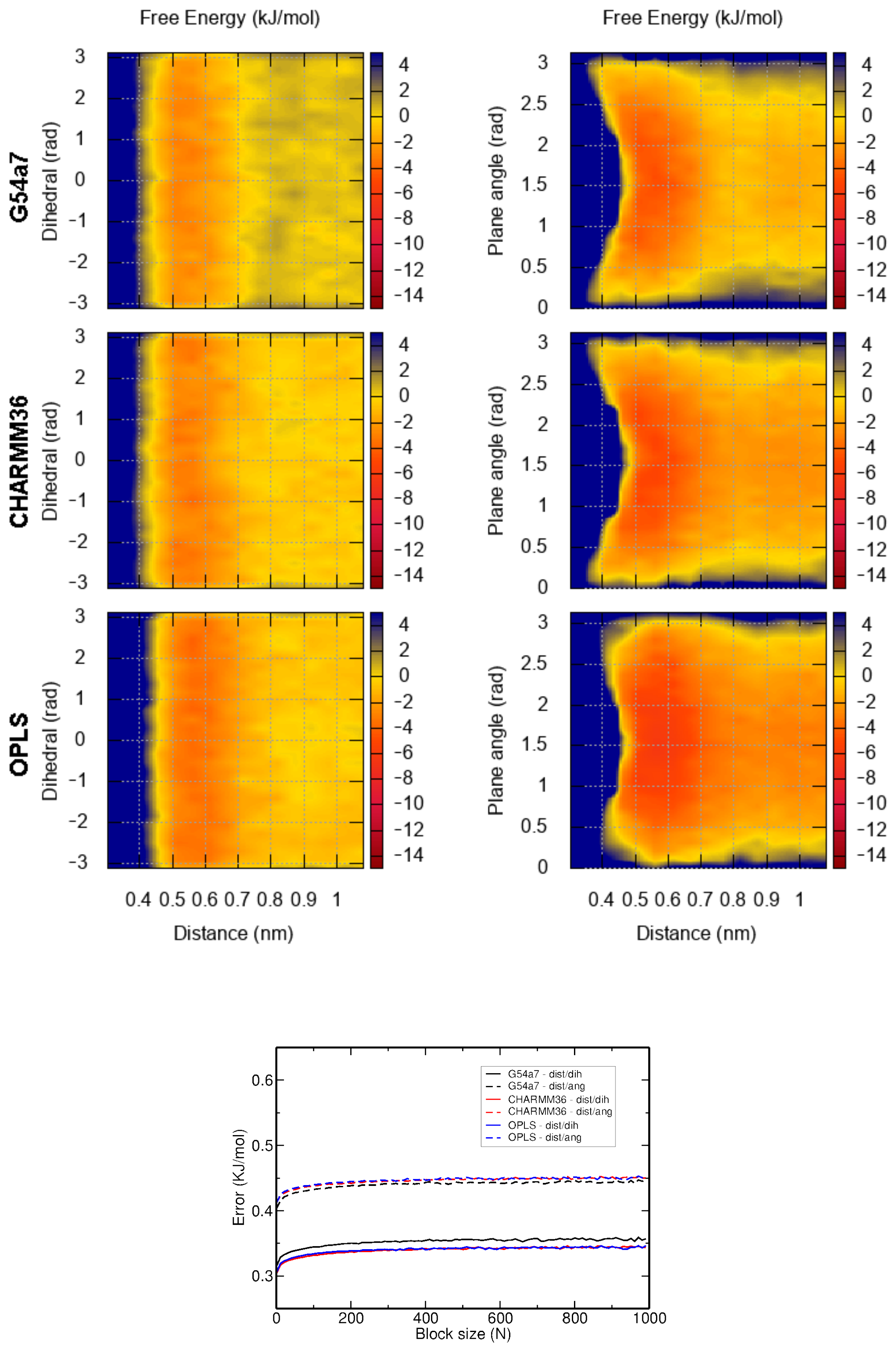

Figure S1: FES for the dimerization of toluene (top) and statistical error of the FES calculations as a function of the block size (bottom).

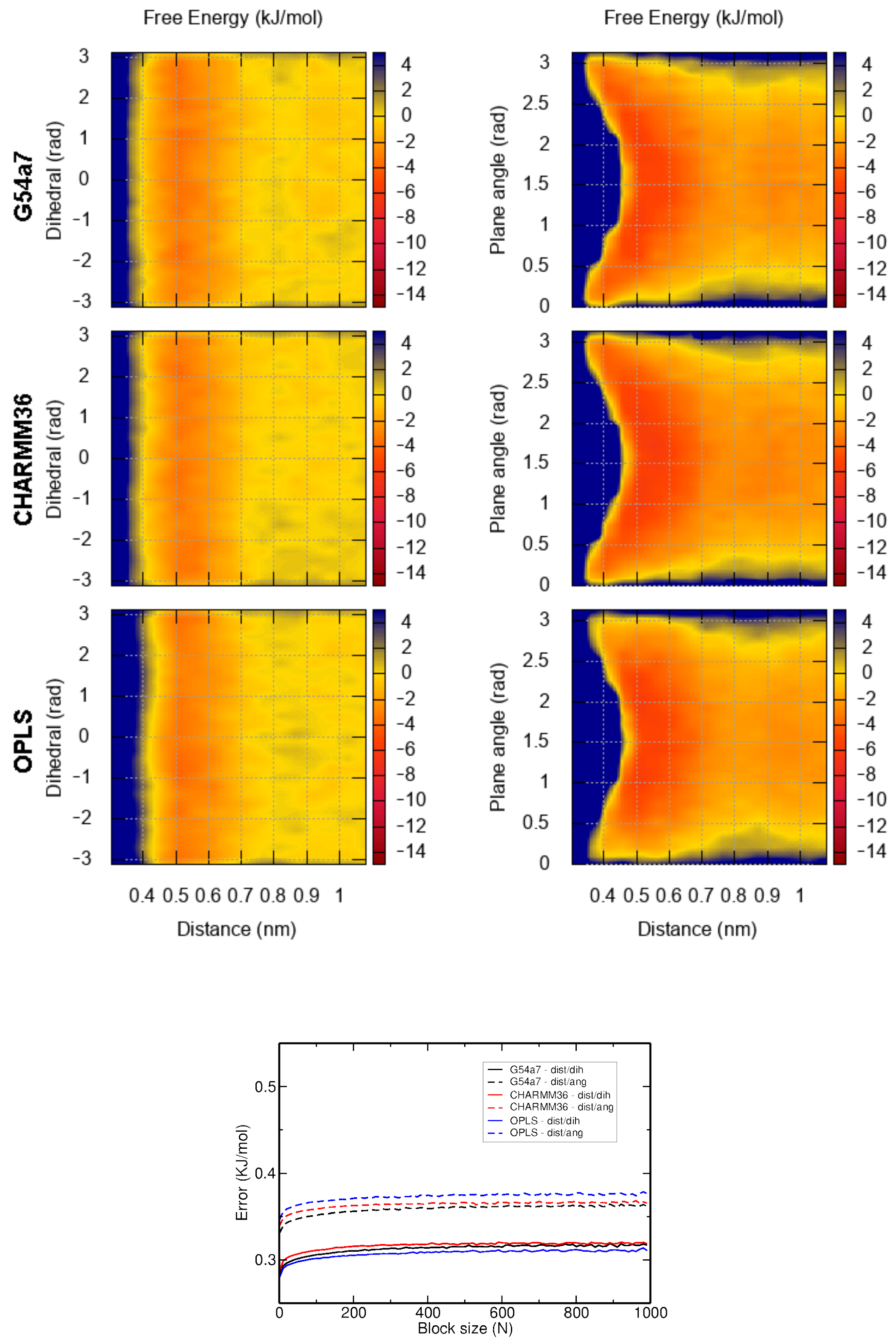

Figure S2: FES for the dimerization of chlorobenzene (top) and statistical error of the FES calculations as a function of the block size (bottom).

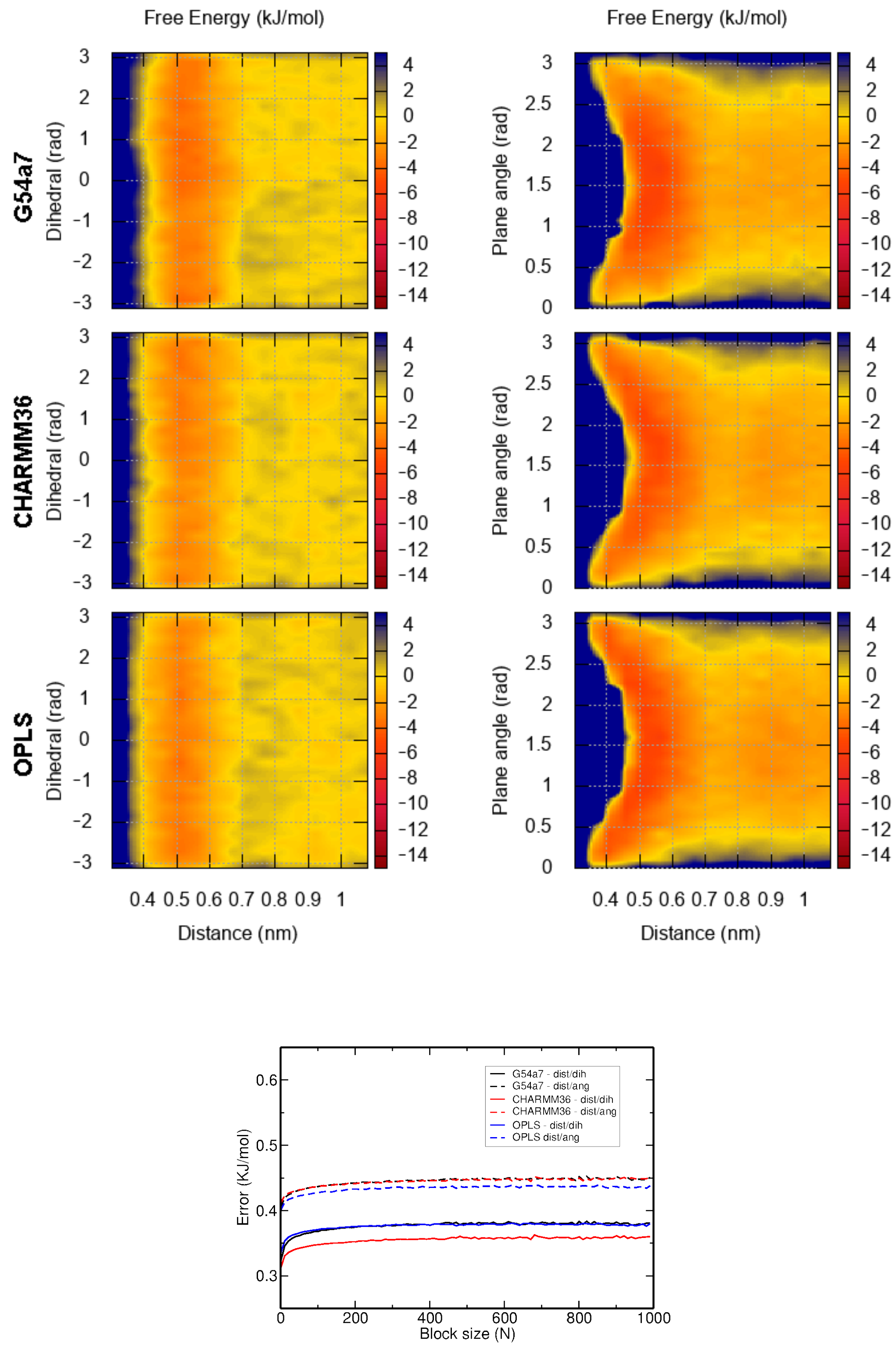

Figure S3: FES for the dimerization of phenol (top) and statistical error of the FES calculations as a function of the block size (bottom).

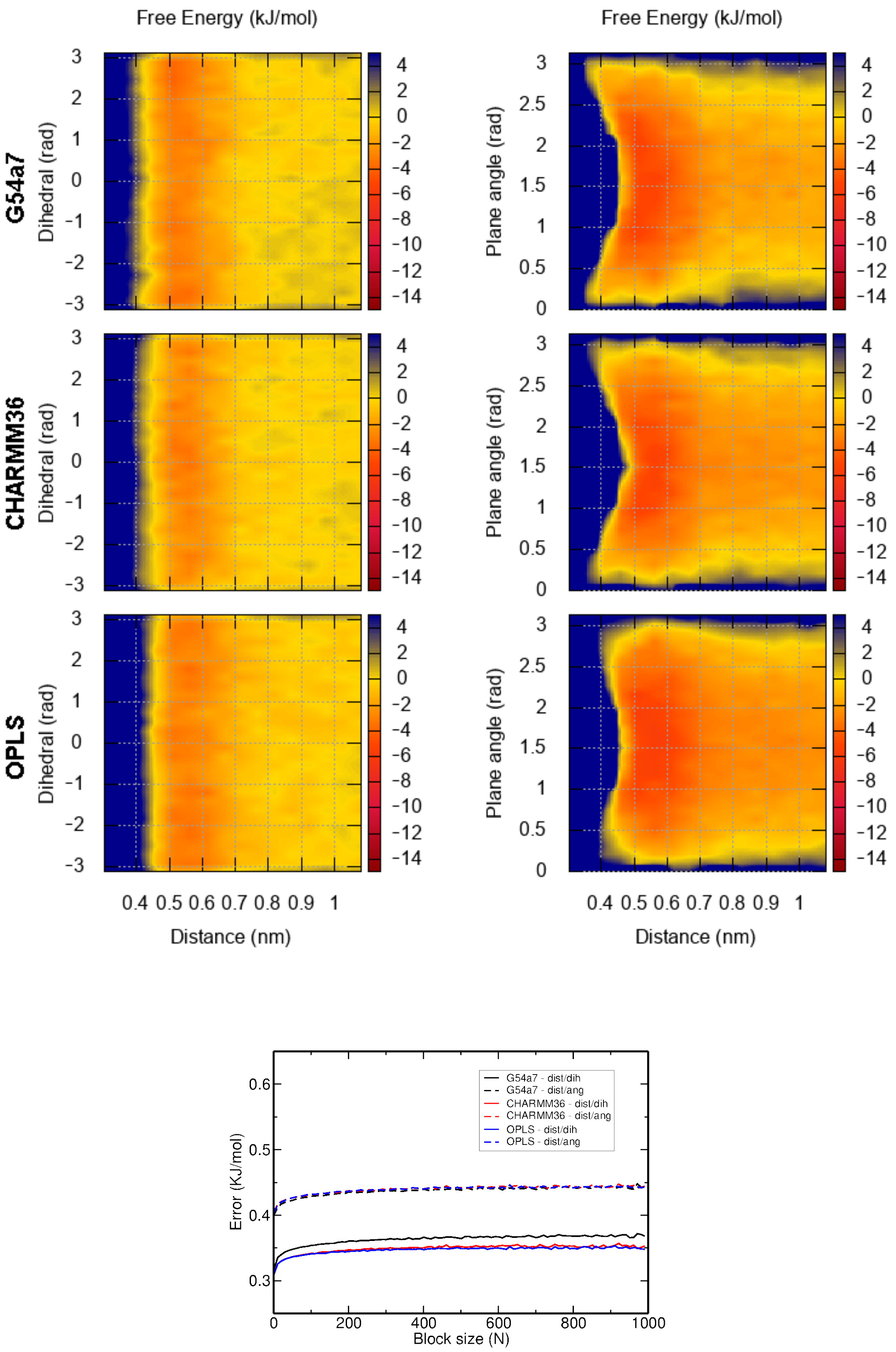

Figure S4: FES for the dimerization of styrene (top) and statistical error of the FES calculations as a function of the block size (bottom).

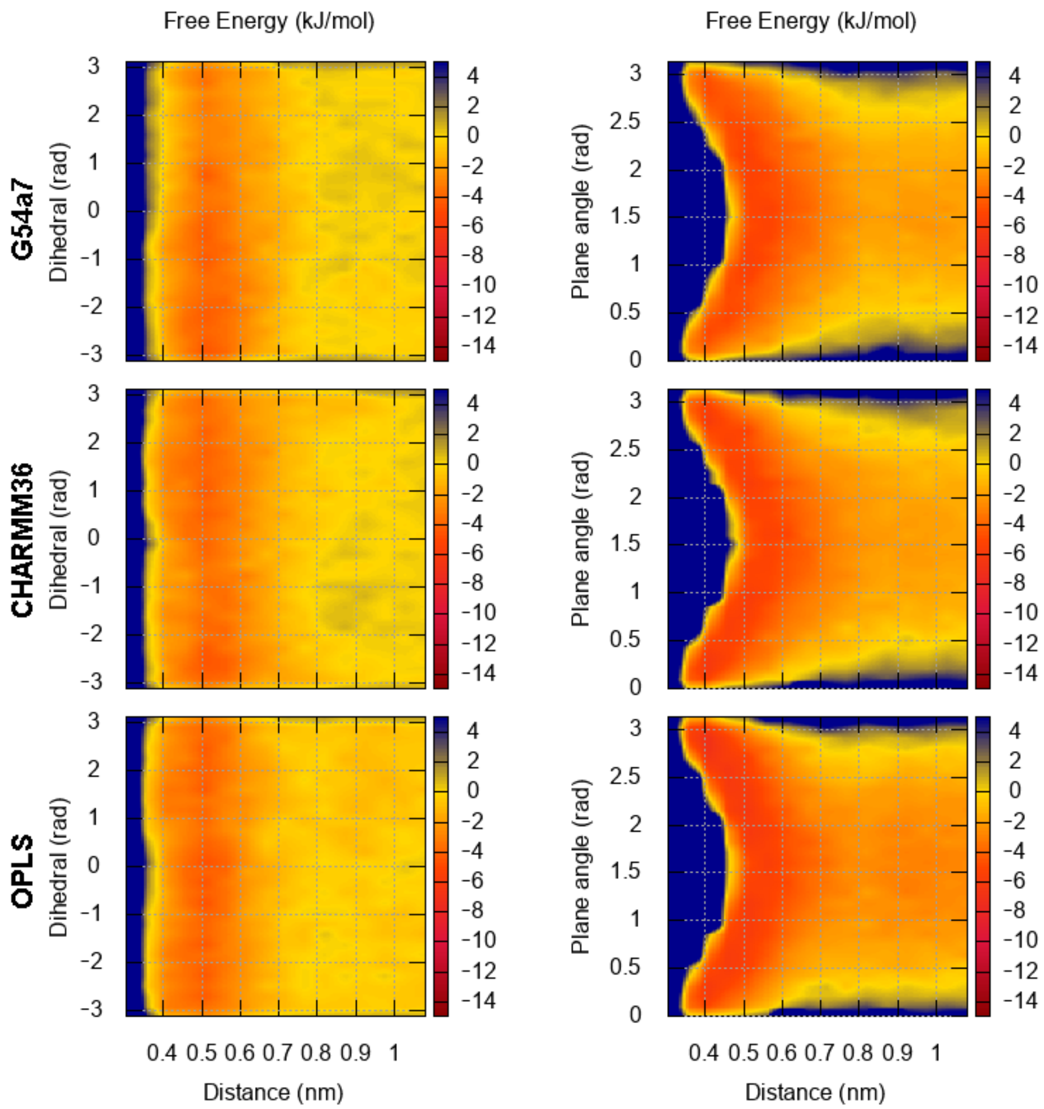

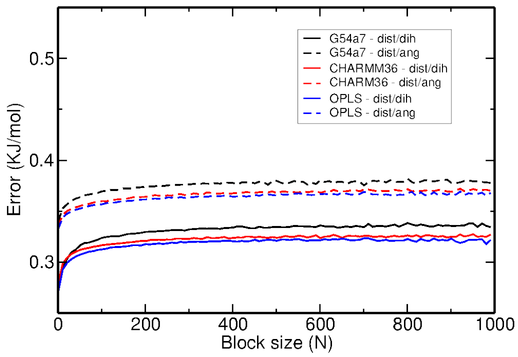

Figure S5: FES for the dimerization of para-cresol (top) and statistical error of the FES calculations as a function of the block size (bottom).

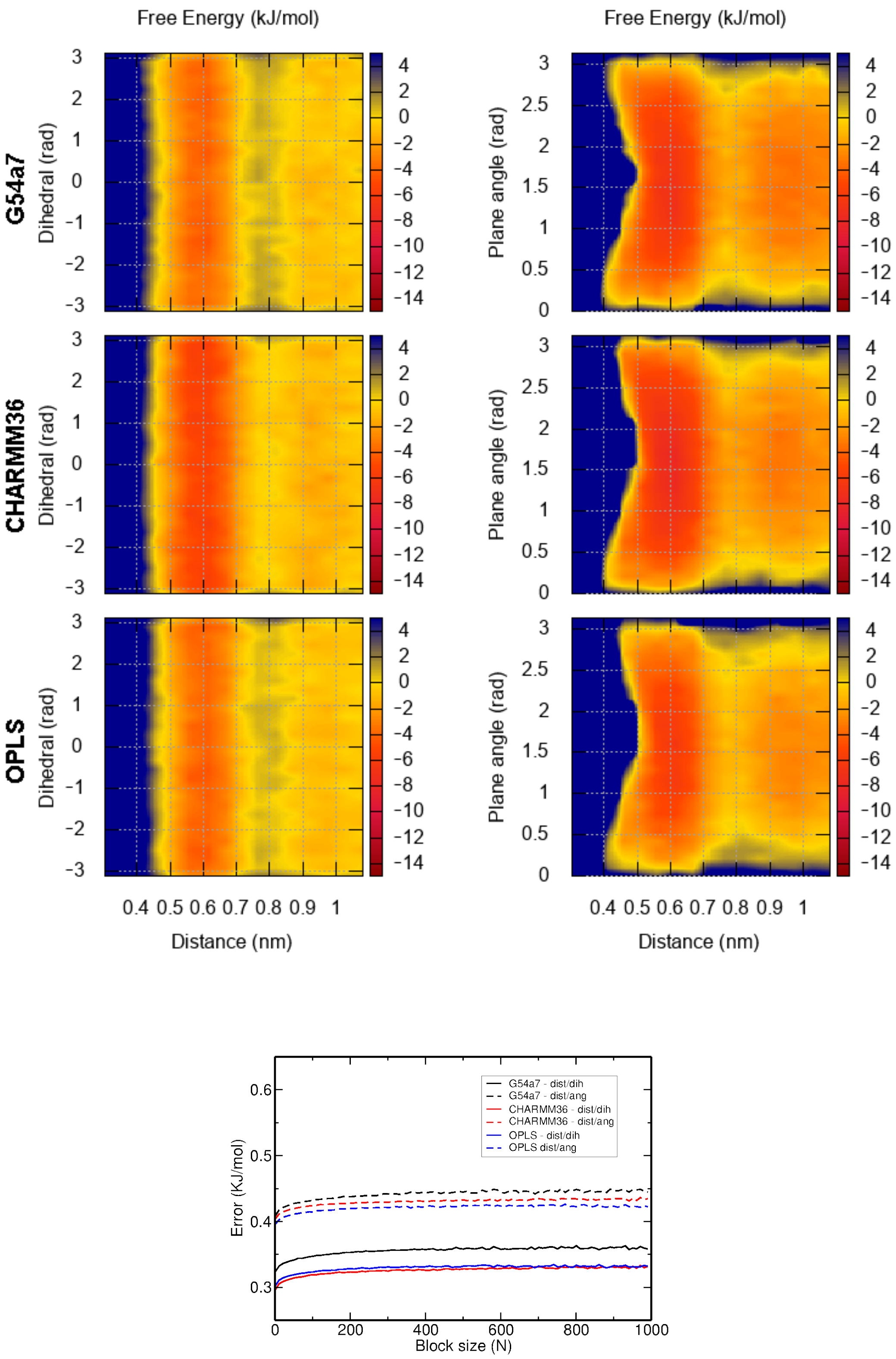

Figure S6: FES for the dimerization of cyclohexane (top) and statistical error of the FES calculations as a function of the block size (bottom).

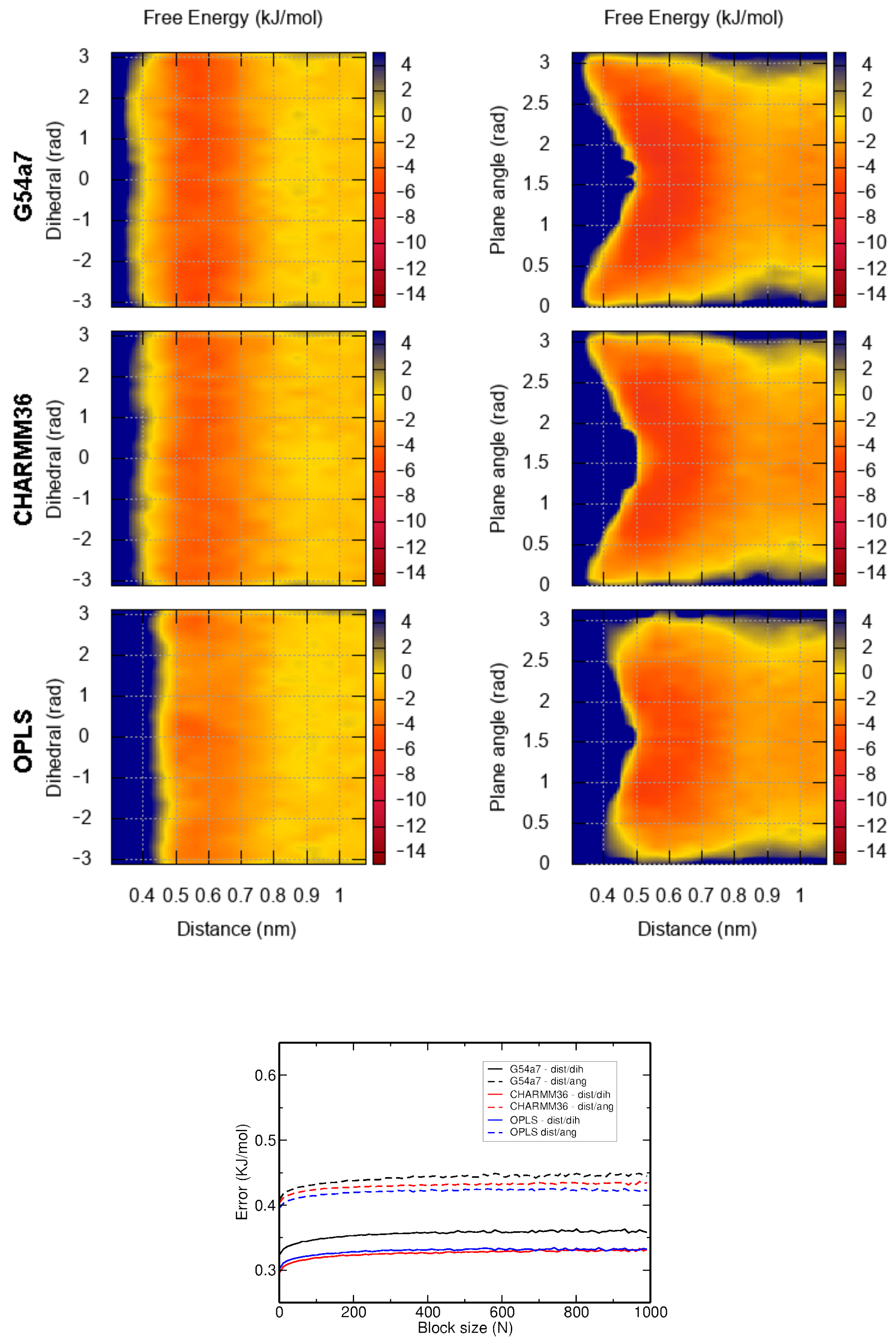

Figure S7: FES for the dimerization of naphthalene (top) and statistical error of the FES calculations as a function of the block size (bottom).

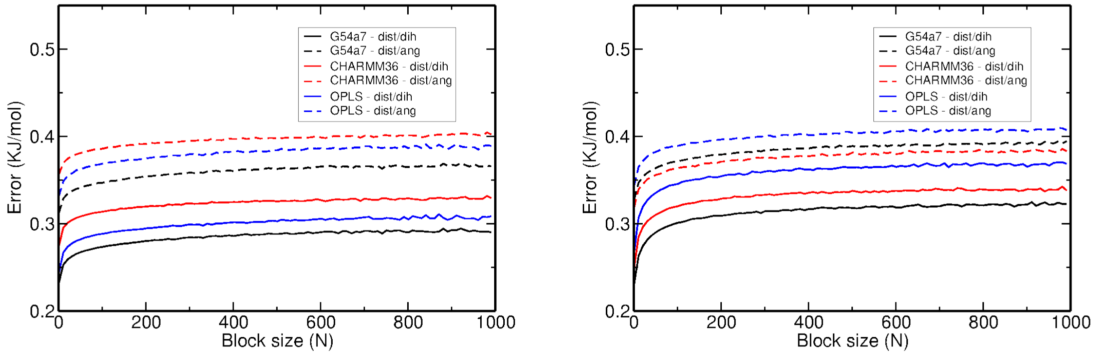

Figure S8: Statistical error of the FES calculations as a function of the block size for pyrene and anthracene, respectively.
